# Evaluation of plasma circ_0006282 as a novel diagnostic biomarker in colorectal cancer

**DOI:** 10.1002/jcla.24147

**Published:** 2021-12-03

**Authors:** Davood Mohammadi, Yazdan Zafari, Zohreh Estaki, Mahdi Mehrabi, Sahar Moghbelinejad

**Affiliations:** ^1^ Department of Surgery School of Medicine Qazvin University of Medical Sciences Qazvin Iran; ^2^ Department of Hematology and Medical Oncology School of Medicine Qazvin University of Medical Sciences Qazvin Iran; ^3^ Department of Pediatric Dentistry School of Dentistry Qazvin University of Medical Sciences Qazvin Iran; ^4^ Student Research Committee Qazvin University of Medical Sciences Qazvin Iran; ^5^ Research Institute for Prevention of Non‐Communicable Diseases Cellular and Molecular Research Centre Qazvin University of Medical Sciences Qazvin Iran

**Keywords:** biomarker, circular RNA, colorectal cancer, hsa_circ_0006282, real‐time polymerase chain reaction

## Abstract

**Background:**

Nowadays, non‐invasive and rapid detection of cancers through molecular biomarkers has received much attention. Therefore, this study investigated the non‐invasive and rapid diagnosis of colorectal cancer through one of the newest biomarkers (circular RNA).

**Methods:**

For this purpose, we collected tumoral, adjacent normal tissue, and plasma samples from 100 colorectal cancer (CRC) patients, 25 postoperative CRC patients, 28 colitis patients, and 108 healthy donors. First Illumina high‐throughput (Hi Seq 2000) sequencing was performed to identify known and novel differentially expressed circRNAs in the cancerous and adjacent normal tissues (n = 3). We used quantitative real‐time fluorescent polymerase chain reaction (qRT‐PCR) to detect the expression level of hsa_circ_0006282 among the different samples. Moreover, inter‐ and intra‐assays were performed to evaluate the potential of hsa_circ_0006282 as being a biomarker. The receiver operating characteristic curve (ROC) was drawn to appraise its diagnostic efficacy, and the sensitivity of this circ RNA was evaluated.

**Results:**

Based on RNA‐sequencing results circ_0006282, cirs7, circ‐0001313, circ_0055625, circ_000984, circ_0055625, circ_0001178, circ_0071589, circ‐001569 were upregulated, and circ‐ITGA7, circ‐CDYL, circITCH, circ_0026344, circ_0000038, circ_0002220, circ_0067480, circIGHV3‐20‐1, circ_104916, circ_0009361 were downregulated circRNA. The hsa_circ_0006282 was the highest upregulated differentially expressed circRNA. Expression evaluation of this circRNA on different samples showed upregulation in CRC tissues (*p *< 0.0001) and plasma samples of CRC patients in comparison to healthy controls (*p *< 0.0001), while the area under the curve (AUC) was 0.831 (95% CI: 0.779–0.883). Expression of hsa_circ_0006282 in CRC patients decreased to normal after surgery (*p *< 0.0001). Our results showed high specificity and sensitivity of CRC detection when hsa_circ_0006282, carcinoembryonic antigen (CEA), and carbohydrate antigen199 (CA199) are combined.

**Conclusion:**

Plasma hsa_circ_0006282 can be used as a novel diagnostic and dynamic monitoring biomarker in CRC patients.

## Graphical abstract

Based on RNA‐sequencing results circ_0006282, cirs7, circ‐0001313, circ_0055625, circ_000984, circ_0055625, circ_0001178, circ_0071589, circ‐001569 were upregulated and Circ‐ITGA7, Circ‐CDYL, CircITCH, Circ_0026344, Circ_0000038, Circ_0002220, Circ_0067480, CircIGHV3‐20‐1, Circ_104916, Circ_0009361 were downregulated circRNA. Upregulation of hsa_circ_0006282 confirmed in CRC tissues and plasma samples of CRC patients in comparison to healthy controls. This expression decreased to normal after surgery. There is a high specificity and sensitivity of CRC detection when hsa_circ_0006282, carcinoembryonic antigen (CEA), and carbohydrate antigen199 (CA199) are combined.

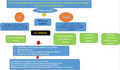

## 1 INTRODUCTION

Colorectal cancer (CRC), also known as bowel cancer, colon cancer, or rectal cancer, is defined as any cancer that affects the colon and rectum. Treatments used for colorectal cancer may include a combination of surgery, radiation therapy, chemotherapy, and targeted therapy. The use of molecular biomarkers in diagnosis of cancers, including colorectal cancer, has great importance.^1^, ^2^ Nowadays, liquid biopsy is used as a non‐invasive diagnostic method in hospital settings.^3^ Currently, carcinoembryonic antigen (CEA) and carbohydrate antigen199 (CA199) are the most used antigens in the diagnosis of gastrointestinal tumors,^4^ but their sensitivity and specificity are low.^5^

Circular RNAs (circRNAs) are a novel class of endogenous noncoding RNAs characterized by their stable closed‐loop structure. They are considered potential disease biomarkers due to their tissue‐ and development stage‐specific expression pattern. Two methods have been proposed to produce these RNAs: (A) back‐splicing and (B) covalent binding.^6^, ^7^ Recently, many studies have been conducted on the function and regulatory mechanisms of circRNAs. In general, the critical role of these RNAs is the gene expression regulation of micro RNAs (miRNAs). Since circRNAs participate in different cell signaling pathways, they could be used as a diagnostic and prognostic aid in various cancers, including human CRC.^6^, ^7^, ^8^ One of the unique features of these RNAs is their covalently closed cyclic structure which makes them resistant to digestion by exonucleases.

For this reason, their expression in cells is stable, and their half‐life is high, particularly in cell‐free samples (such as blood, urine, and saliva).^9^ Human hsa_circ_0006282 is one of the newly introduced circRNAs with 198bp nt in spliced sequence length. Its gene is located at chr8:74868145–74872053, and its gene symbol is TCEB1.^10^ Our study aimed to evaluate the diagnostic efficacy and sensitivity of tissue and plasma hsa_circ_0006282 for early and rapid diagnosis of CRC.

## 2 MATERIALS AND METHODS

### 2.1 Collection of plasma and tissue samples

This study was approved by the Ethics Committee of Qazvin University of Medical Sciences, and the consent forms were studied and signed by each patient. The study protocol conforms to the Declaration of Helsinki. In this study, 100 samples of cancerous tissue and 100 adjacent normal tissue were taken from the CRC patients; all patients had adenocarcinoma of the rectum or colon. This sampling was done in the Surgical Center of Velayat Hospital in Qazvin and Shariati Hospital in Tehran, Iran, during the 2019–2020 years. After taking the tissue samples, they were stored at −80°C immediately to prevent RNA degradation. The plasma samples included 100 CRC patients, 25 postoperative CRC patients, 28 cases of colitis patients, and 108 cases of age‐matched healthy controls. In our research, all CRC patients were diagnosed as primary by clinicians and did not receive chemotherapy or radiotherapy before the surgery. Patients’ documents have been carefully reviewed, and their pathological information was also collected.

### 2.2 Cell culture

NCM460 as normal human colon epithelial cells and 3 CRC cell lines (SW480, SW620, and Lovo) were purchased from Cell Bank of Pasture Institute (Tehran, Iran). The cells were incubated in Roswell Park Memorial Institute‐1640 medium (RPMI‐1640) (Sigma‐Aldrich) and supplemented with 10% fetal bovine serum. Cells were cultured under standard conditions and incubated at 37°C and 5% CO_2_.

### 2.3 Circular‐RNA sequencing and quantitative real‐time reverse transcription‐polymerase chain reaction (qRT‐PCR)

For total RNA extraction, 700 mg of the frozen tissue, 1 × 10^6^ of the cultured cells, and 350 µl of plasma were prepared, then 1 ml TRIZOL reagent (Invitrogen) was added to the centrifuged cells following the manufacturer's instruction. To evaluate the quantity and quality of extracted RNA, we used NanoDrop 2000c (Thermo). In this case, RNA samples with A260/A280 ratios of >2 were selected for sequencing and quantitative analysis. RNase R was used for the treatment of total RNA and to improve the purity of circRNAs. First‐strand complementary DNA (cDNA) synthesis was also performed on the treated RNA using the Revert Aid First Strand cDNA Synthesis Kit (Thermo Scientific, Fermentas). First, the RNA samples were size‐fractionated, then 18–30 nt RNA was isolated and purified. 5′ and 3′ adaptor ligation was done for cDNA synthesis. PCR situation for cDNA synthesis was 98°C for 10 s and 72°C for 15 s in 14 cycles. The Agilent technologies 2100 Bioanalyzer was used to check the size (average molecule length) and purity, and quantitative PCR using Eva Green dye (Jena Bioscience) was used to verify the concentration. Then sequencing was done on Illumina HiSeq 2000 (Illumina, Inc.). After scoring the top upregulated and downregulated circRNAs, hsa_circ_0006282 was selected as the highest upregulated circRNA for other evaluations.

Hsa_circ_0006282 expression was evaluated using quantitative real‐time polymerase chain reaction (qRT‐PCR) (Rotor‐gene). It should be noted that we used GAPDH as the internal control. Our primer sequences were: hsa_circ_0006282: Forward, 5ʹ‐AGGCACGATAAAAGCCATGT‐3ʹ (forward) and 5ʹ‐GGTCCTTCACAGCCACCATA‐3ʹ (reverse), GAPDH: GAAGGTGAAGGTCGGAGTC (forward) and GAAGATGGTGATGGGATTTC (reverse).^11^

The reactions were incubated in a 72‐well optical strip at 95°C for 15 min (enzyme activation), followed by 95°C for 20 s and 60°C for 60 s (40 cycles). All reactions were run in triplicate. After these reactions, the mean Ct was determined from the triplicate PCRs. We used Ct values to evaluate the expression levels of the hsa_circ_0006282. The expression value of the mentioned CircRNA relative to internal control was determined using the 2^−△Ct^ method.^12^

### 2.4 Statistical analysis

The results were analyzed by GraphPad software (GraphPad PRISM V 8.4 analytical software). We used Student's t test and Pearson's χ^2^ test, respectively, to compare data between pairs of groups and to evaluate the clinicopathological variables.

## 3 RESULTS

### 3.1 Expression profile of circRNAs in tissue samples

Three pairs of CRC tissues and adjacent normal tissues were used for expression profile study. Our results showed that 4870 circRNAs were expressed differently in CRC tissues in comparison to normal ones. According to the principle of expression difference multiple >2.0 and *p* < 0.05, 258 circRNAs were upregulated, and 145 circRNAs were downregulated in CRC patients. The lists of top upregulated and downregulated CircRNAs are given in Tables 1 and 2. By evaluating different databases such as CircRNA Db, CircBase, and CircBank, hsa_circ_0006282 was selected for subsequent studies.

**TABLE 1:** Top upregulated circular RNAs in cancerous tissue samples of colorectal cancer patients in comparison to adjacent normal ones

CircRNA	No. of reads	2^−∆ct^	*p*‐value
Circ_0006282	89.011/213.321	10.213	0.00001
Cirs7	9.0021/4.21	9.7653	0.00015
Circ−0001313	7.321/1.991	6.123009	0.000121
Circ_0055625	104.10/1.32	5.00043	0.0032
Circ_0000284	100.08/154	3.8754	0.0103
Circ_000984	108.11/124	3.0043	0.01354
Circ_0055625	42.01/102	1.76544	0.0113
Circ_0001178	5/102	1. 7003	0.00123
Circ_0071589	9/001	1.7735	0.011
Circ−001569	9/012	1.5023	0.0001

**TABLE 2:** Top downregulated circular RNAs in cancerous tissue samples of colorectal cancer patients in comparison to adjacent normal ones

CircRNA	No. of reads	2^−∆ct^	*p*‐value
Circ‐ ITGA7	65.12/321.121	0.00042	0.00002
CircITCH	14.123/4.65	0.00038	0.000016
Circ_0026344	6.321/1.991	0.00021	0.000132
Circ_0000038	604.9/1.32	0.00013	0.0032
Circ_0002220	89.12/453	0.0023	0.0103
Circ_0067480	234/124	0.0021	0.013
CircIGHV3‐20‐1	34/102	0.0019	0.012
Circ_104916	11/102	0.0011	0.0016
Circ_0009361	10/006	0.013	0.011
Circ‐CDYL	8/011	0.12	0.009

### 3.2 The expression rate of hsa_circ_0006282 in different samples

To select the appropriate internal control in real‐time PCR method, gene expression of 18S rRNA, GAPDH, β‐actin, and β2M (Microglobin) were evaluated in 25 CRC patients and 25 healthy controls. Finally, GAPDH showed stability in the studied samples and was selected as the internal control.

**FIGURE 1:**
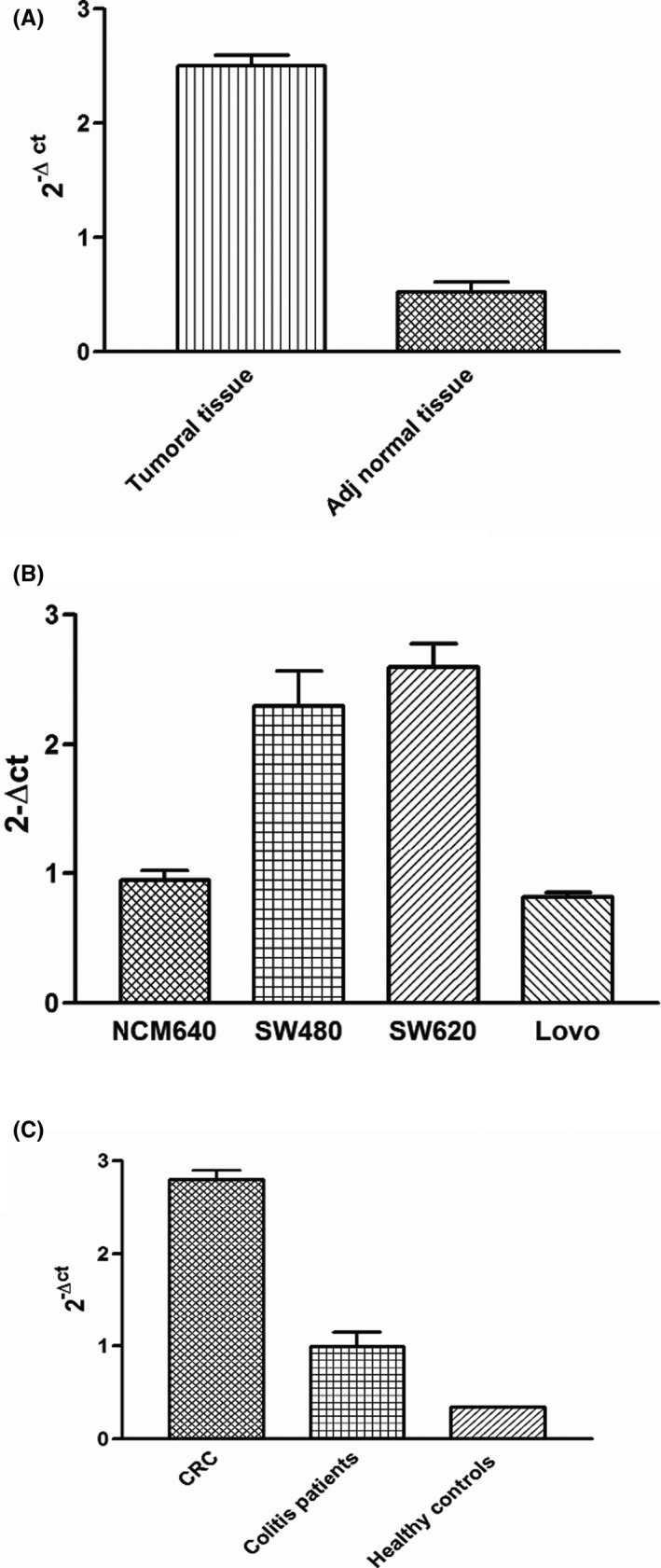
(A) Overexpression of hsa_circ_0006282 in cancerous tissues in comparison to adjacent normal tissues (*p* < 0.0001), (B) overexpression of hsa_circ_0006282 in SW480, SW620 cell lines in contrast to normal epithelial cells (*p* < 0.0001), (C) differential expression of hsa_circ_0006282 in the plasma of CRC patients (n = 100), colitis patients (n = 28), and healthy donors (n = 108), our results showed overexpression of hsa_circ_0006282 in plasma samples of CRC patients compared to colitis patients (n = 28) and healthy donors (n = 108) (*p* < 0.001)

The expression rate of hsa_circ_0006282 was also studied in 100 CRC and adjacent normal tissues. Our results showed upregulation of this circRNA in CRC tissues when compared to the adjacent normal tissues (*p* < 0.0001) (Figure 1A). Moreover, results regarding the expression level of hsa_circ_0006282 in CRC cell lines (SW480, SW620, Lovo) and NCM460 as normal human colon epithelial cells showed significant upregulation of hsa_circ_0006282 in SW480, SW620 cell lines in comparison to normal epithelial cells (*p* < 0.0001) (Figure 1B).

**FIGURE 2:**
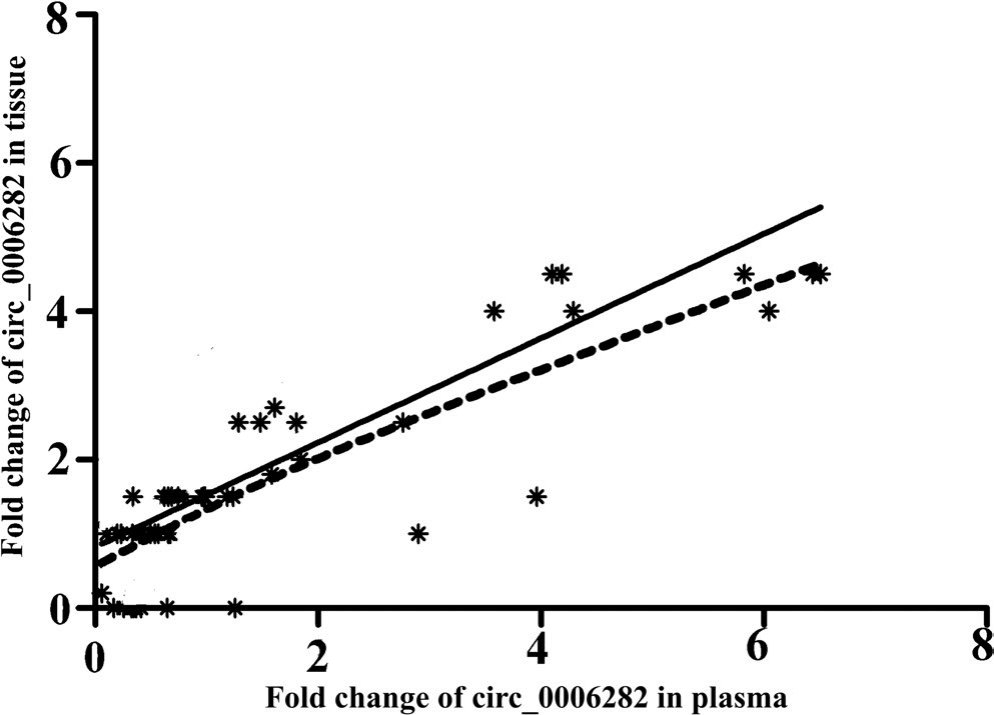
positive correlation of the relative expression of hsa_circ_0006282 between plasma and tissues in GC patients (*p* < 0.01, R^2^ = 0.6706)

In recent years, liquid biopsy has received a great deal of attention from researchers, and the exciting point is that circRNAs can be detected in human body fluids. In this research, we evaluated the potential of hsa_circ_0006282 as a plasma biomarker in CRC. The results of the expression rate evaluation of hsa_circ_0006282 in plasma samples of CRC patients showed a significant high expression rate of this circRNA in comparison to the healthy control group and colitis patients (*p* < 0.001); however, there was no significant difference in the expression level of hsa_circ_0006282 between colitis patients and controls (*p* = 0.12) (Figure 1C). Also, we evaluated the expression level of hsa_circ_0006282 in patients after surgery; our results showed that the expression rate of plasma hsa_circ_0006282 decreased to normal immediately after surgery and increased after recurrence in comparison to normal plasma samples (*p* < 0.0001). Similarly, the Pearson correlation test result showed a significant positive correlation between the expression rate of tissue and plasma samples in CRC patients (R = 0.563 *p* < 0.022) (Figure 2).

**FIGURE 3:**
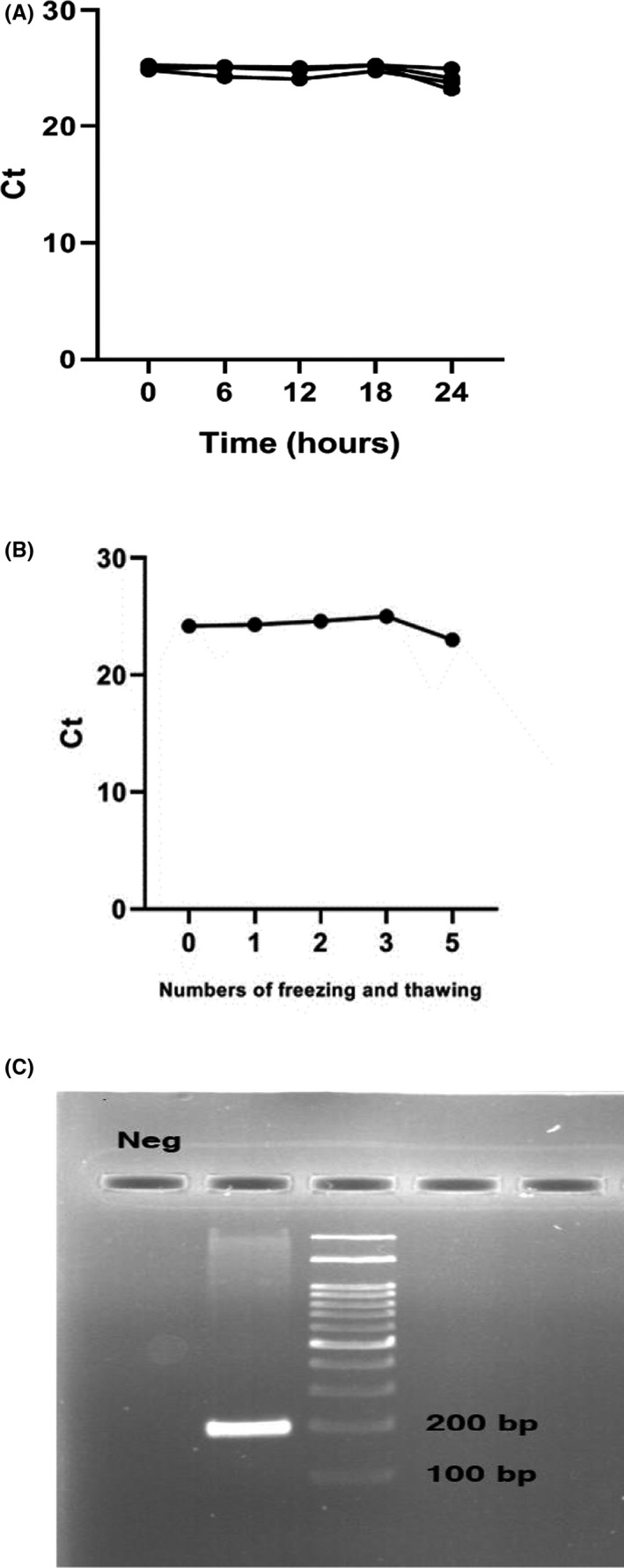
(A) Intra‐assay and (B) Inter‐assay coefficient variation of hsa_circ_0006282 detection (*p *> 0.05). (C) Shows the accuracy and specificity of hsa_circ_0006282 by Agarose gel electrophoresis

To evaluate the repeatability and stability of this circRNA, a mix of plasma was provided and was placed at room temperature for 0, 2, 6, 10, 14, and 24 h. Then, mixed plasma was frozen and thawed for 0, 1, 2, 3, and 5 times, the relative expression rate of hsa_circ_0006282 was detected. Based on our results, there was no significant difference in the expression rate of hsa_circ_0006282 in plasma between different examined periods (*p *> 0.05) (Figure 3A,B), which indicated that the detection of hsa_circ_0006282 had good repeatability and stability. Finally, we detected an amplification product of qRT‐PCR on the agarose gel (198bp) (Figure 3C).

### 3.3 Diagnostic utility of plasma Circ_0006282 in CRC

**FIGURE 4:**
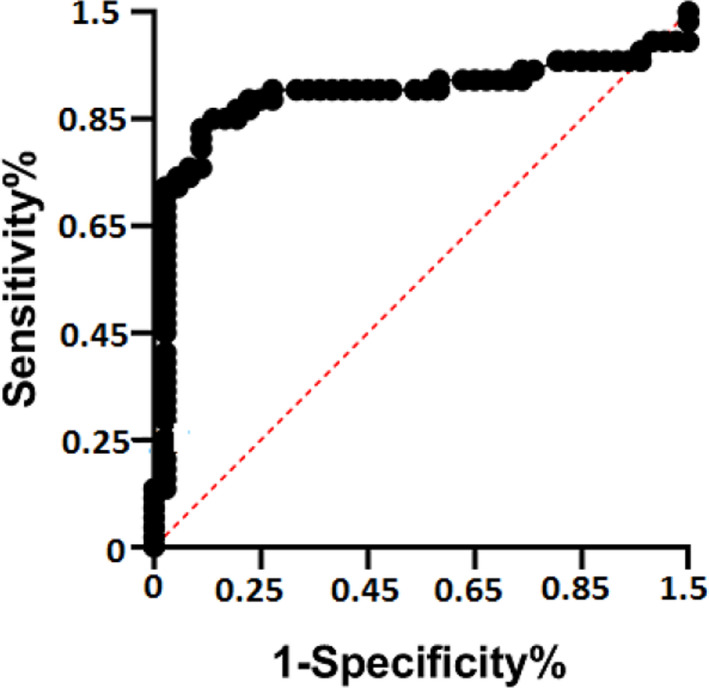
ROC curve analysis of plasma hsa_circ_0006282 for discriminating primary CRC patients and healthy donors (AUC = 0.812 (95% CI: 0.625–0.801, *p* < 0.0001))

The receiver operating characteristic curve (ROC) and area under curve (AUC) were drawn for hsa_circ_0006282 plasma levels of 100 patients and 108 age‐matched control to evaluate hsa_circ_0006282 as a biomarker for CRC diagnosis. Our results showed that plasma hsa_circ_0006282 could distinguish the primary CRC patients from the healthy samples, and the AUC was 0.812 (95% CI: 0.625–0.801, *p* < 0.0001) (Figure 4). Based on Table 3 results, combining hsa_circ_0006282, CEA, and CA199 could improve the diagnostic sensitivity (78.8%), while the sensitivity of CEA and CA199 alone are 48.3% and 29%, respectively. Regarding the clinicopathological characteristics of 100 CRC patients, we showed that the high expression level of plasma hsa_circ_0006282 is associated with poor differentiation (*p* = 0.012), lymph node metastasis (*p* < 0.001), T stage (*p* < 0.0001), and TNM stage (*p* < 0.01) (Table 4). All of the above data indicated that plasma hsa_circ_0006282 has high diagnostic efficiency.

**TABLE 3:** Assessment of the diagnostic values of combination of hsa_circ_0006282, CEA, CA199 between CRC patients and controls. SEN: sensitivity, SPE: specificity, ACCU: overall accuracy, PPV: positive predictive value, NPV: negative predictive value

	SEN, %	SPE, %	ACCU, %	PPV, %	NPV, %
Hsa_circ_0006282	71.2	80.1	75	74.1	68.1
CEA	48.3	77.2	59	69.2	59.3
CA19‐9	29.1	77.8	53.1	59	58.1
Hsa_circ_0006282+CEA	79.3	77.8	80	75.2	78.3
Hsa_circ_0006282+CA19‐9	63.8	88.1	79.1	76.3	69.1
Hsa_circ_0006282+CA19‐9+CEA	78.8	76.9	79	74.2	79

**TABLE 4:** Correlation of hsa_circ_0006282 expression with "cilinicopathological" parameters. T: refers to the size and extent of the main tumor, TNM: number to describe the tumor (T), node (N), and metastasis (M)

Parameters	hsa_circ_0006282 expression in tumor		*p‐*value
	Low	High	
Gender			
Male	32	35	
Female	19	14	0.12
Age			
<55 years	16	8	0.55
≥55 years	46	30	
Tumor size			
<5 cm	9	15	0.72
≥5 cm	50	26	
Differentiation			
Well+moderate	18	15	**0.012**
Poor	17	50	
T classification			
1–2	30	28	**<0.0001**
3–4	12	30	
Lymph node metastasis			
Positive	25	20	**<0.001**
Negative	15	40	
TNM stage			
I–II	30	22	**<0.01**
III–IV	13	35	
CEA			
Positive	25	30	0.54
Negative	27	18	
CA19‐9			
Positive	28	20	0.65
Negative	24	28	

Note

The bold value means statistical significance.

## 4 DISCUSSION

Colorectal cancer is one of the most common cancers with a high mortality rate, and most cases of CRC patients are found at an advanced stage.^13^ Although there are different biomarkers for detecting colorectal cancer, these biomarkers are not very sensitive and specific. Today, the use of molecular biomarkers in the early detection of cancers is very crucial. CircRNAs are among these biomarkers that have been received too much attention in the last three or four years.^14^, ^15^, ^16^

In this regard, Chiu et al. showed that 10,245 CircRNAs were expressed aberrantly in CRC patients, 3981 downregulated, and 6264 upregulated.^17^ Also, Zhu et al. investigated the expression of circRNAs by using a circRNA microarray. They chose the most significantly upregulated circRNA, hsa_circ_0007142, for their study and found that hsa_circ_0007142 was associated with lymphatic metastasis and differentiation of CRC.^18^

In this study, we examined hsa_circ_0006282. For this purpose, the expression level of this circRNA in tissue and plasma samples of patients with CRC was investigated. Considering a significant positive relationship between expression in tissue and plasma samples, we concluded that this circRNA is released from tumor tissue cells and enters the plasma.

The plasma expression rate of hsa_circ_0006282 in CRC patients was significantly higher than healthy controls and samples with colitis. Also, our ROC analysis indicated that hsa_circ_0006282 could well distinguish CRC patients from healthy controls and colitis patients. Additionally, the sensitivity and specificity of this biomarker in combination with CEA and CA199 were evaluated. Its sensitivity and specificity were 78.8% and 76.9%, respectively. When we evaluated the expression level of hsa_circ_0006282 in patients after surgery, our results showed that the expression rate of plasma, decreased to normal immediately after surgery and increased after recurrence; this suggests that plasma hsa_circ_0006282 can be used as a dynamic indicator.

In recent years, various studies have been conducted on plasma circRNAs as a biomarker in cancer diagnosis.^10^, ^11^, ^12^, ^13^, ^14^, ^15^, ^16^, ^17^, ^18^, ^19^, ^20^, ^21^ Dao‑Xiong et al. introduced three plasma circRNAs (circ_0082182, hsa_circ_0000370, and hsa_circ_0035445) that can be used as biomarkers for the detection of CRC.^22^ Yung‐Hung et al. reported upregulation of hsa_circ_0000190 in plasma samples of patients with lung cancer.^23^ Generally speaking, circRNAs can be released from cancer cells into plasma, from dead cancer cells in exosome form, or by deregulation of cancer cells matrix structure under the influence of drugs.^24^, ^25^ Regarding the role of hsa_circ_0006282 in cancer incidence, only one study was conducted in 2020, which showed an increase in the expression of hsa_circ_0006282 in patients with gastric cancer.^11^They also suggested that the oncogenic function of circ_0006282 is partly attributed to its regulation on miR‐155/FBXO22 axis by sponging miR‐155 to upregulate the expression of FBXO22. Since one of the essential roles of the circRNAs is the sponge of miRNAs, in the future study, we will investigate the circRNA‐miRNA‐mRNA axis in CRC patients.

In conclusion, plasma hsa_circ_0006282 can be used as a novel biomarker in the progression of CRC. Plasma hsa_circ_0006282 has the potential to be used as an early screening indicator in the detection of primary CRC. Given that, after surgery or recurrence, the expression level of plasma hsa_circ_0006282 has dynamically changed, suggesting that this circRNAs may have a real‐time monitoring function. Furthermore, the differential expression of hsa_circ_0006282 in plasma samples of CRC and colitis patients showed that this biomarker could differentiate between CRC and colitis patients.

## CONFLICT OF INTEREST

The authors declare that there is no conflict of interest.

## Data Availability

The data that support the findings of this study are available on request from the corresponding author [SM]. The data are not publicly available because they contain information that could compromise the privacy of research participants.
